# Spectral computed tomography characterization of coronary atherosclerotic plaque: principles, imaging biomarkers, and clinical significance

**DOI:** 10.3389/fcvm.2026.1828158

**Published:** 2026-05-29

**Authors:** Yujian Liu, Yu Peng, Hao Wu, Yuan Li

**Affiliations:** 1 Department of Radiology, Zigong First People’s Hospital, Zigong, Sichuan, China; 2 Department of Radiology, The First Affiliated Hospital of Chongqing Medical University, Chongqing, China; 3 Department of Ultrasound, Zigong Fourth People’s Hospital, Zigong, Sichuan, China

**Keywords:** CCTA, coronary plaque, imaging biomarkers, photon-counting CT, risk stratification, spectral CT

## Abstract

**Background:**

Coronary atherosclerotic plaque vulnerability is determined not only by luminal stenosis but also by plaque composition, calcification pattern, inflammatory activity, and local microenvironmental changes. Conventional coronary computed tomography angiography (CCTA) is well established for anatomic assessment, but its ability to characterize plaque remains limited by the single-energy imaging framework.

**Purpose:**

To review the technical basis, imaging biomarkers, and clinical significance of spectral computed tomography (CT), including photon-counting computed tomography (PCCT), for coronary plaque characterization.

**Content:**

Spectral CT introduces multi-energy information and material decomposition, thereby extending plaque assessment beyond single-Hounsfield unit-based interpretation. Recent studies suggest three major advances: improved plaque composition characterization through energy-dependent attenuation patterns and material-specific analysis; improved structural fidelity in calcified lesions through higher spatial resolution and reduced blooming, particularly with ultrahigh-resolution PCCT; and extraction of surrogate signals of selected pathobiologic processes through iodine-related metrics and emerging molecular imaging approaches. Current evidence indicates that the most consistent clinical value of spectral CT lies in more accurate assessment of calcified lesions, more reproducible quantification of high-risk plaque burden, and improved downstream decision-making through higher specificity and better positive referral yield. However, evidence remains insufficient to establish robust incremental value for long-term prognostic prediction, and important barriers persist, including cross-platform inconsistency, lack of quantitative standardization, limited scanner availability, radiation-dose considerations, post-processing burden, and limited multicenter validation.

**Conclusion:**

Spectral CT is reshaping noninvasive coronary plaque assessment by improving structural fidelity, compositional characterization, and quantitative reproducibility. Its current role is best understood as a noninvasive structural-compositional imaging platform with complementary value relative to intravascular and functional imaging modalities. Broader clinical translation will depend on hardware refinement, quantitative standardization, workflow integration, and prospective validation within unified risk assessment frameworks.

## 1 Introduction

Coronary atherosclerotic heart disease remains one of the leading causes of death and disability worldwide (1–4). With ongoing advances in medical imaging and the widespread use of coronary computed tomography angiography (CTA) in the evaluation of coronary artery disease, the focus of imaging has gradually shifted from simple assessment of stenosis severity to the biological heterogeneity of the plaque itself (5). A large body of clinical evidence indicates that acute cardiovascular events are not determined solely by luminal stenosis, but are also closely related to plaque composition, patterns of calcification, inflammatory activity, and changes in the local microenvironment (5–7). Accordingly, how to characterize coronary plaque more accurately under noninvasive conditions has become a major topic in contemporary cardiovascular imaging (7, 8).

Conventional coronary CTA has well-established strengths in depicting coronary anatomy and assessing stenosis, but its ability to characterize plaque remains constrained by the single-energy imaging framework (9). Classification methods based on a single Hounsfield unit (HU) value show substantial overlap among lipid-rich tissue, fibrous tissue, and mild calcification, and are influenced by tube voltage, reconstruction algorithms, and calcium-related blooming artifacts (10, 11). As a result, CCTA remains limited for quantitative plaque composition assessment, control of calcification-related bias, and identification of surrogate signals of underlying pathobiologic processes (8).

By introducing multi-energy information and material decomposition, spectral CT offers a technical approach to coronary plaque assessment that differs fundamentally from conventional CCTA (11). In recent years, studies of dual-energy/spectral CT and photon-counting CT have suggested potential advantages in plaque composition characterization, reconstruction of calcified plaque boundaries, quantification of low-attenuation components, and extraction of surrogate signals related to selected pathobiologic processes (11, 12). Improvements in spatial resolution and spectral separation with PCCT have drawn particular attention for more faithful structural assessment in heavily calcified lesions (12, 13). However, the true incremental value of these techniques for risk stratification, prognostic assessment, and clinical translation remains incompletely synthesized (12, 14).

Against this background, the present review focuses on the spectral CT characterization of coronary atherosclerotic plaque. We discuss its technical foundations, recent advances in plaque identification, correlations with histopathologic features, clinical significance in risk stratification, and complementary roles relative to other cardiovascular imaging modalities. This narrative review synthesizes representative clinical, technical, and methodological studies using a thematic framework focused on technical principles, imaging biomarkers, clinical implications, translational barriers, and future standardization needs. Our aim is to provide a clearer framework for the role of spectral CT in the precise assessment of coronary plaque.

For this narrative review, we performed a structured literature search of PubMed/MEDLINE, Web of Science, and Scopus for English-language articles published mainly from January 2020 to the time of manuscript preparation. Search terms included combinations of “spectral CT,” “dual-energy CT,” “photon-counting CT,” “coronary CT angiography,” “coronary plaque,” “atherosclerotic plaque,” “low-attenuation plaque,” “iodine,” “effective atomic number,” “K-edge imaging,” “calcified plaque,” and “blooming artifact.” Key earlier methodological, technical, and consensus papers were also included when they provided essential background or clinical context. Articles were prioritized if they addressed technical principles, plaque characterization, histopathologic or intravascular imaging correlation, diagnostic performance, reproducibility, clinical workflow, or translational limitations. This review was not designed as a systematic review or meta-analysis, and no formal PRISMA-based screening or quantitative evidence synthesis was performed.

## 2 Technical principles and imaging modes of spectral CT

### 2.1 Physical basis of multi-energy imaging and material decomposition

Within the diagnostic energy range, the interaction of x-rays with matter is governed primarily by the photoelectric effect and Compton scattering. The photoelectric effect is highly sensitive to atomic number, whereas Compton scattering more strongly reflects electron density. Different tissues and materials therefore exhibit systematic differences in attenuation curves across energy levels, providing the physical basis for material separation and quantitative analysis in spectral CT (11, 15, 16).

By acquiring attenuation information at two or more energy levels, the attenuation characteristics within a voxel can be decomposed into linear combinations of several basis materials, thereby enabling quantitative estimation of material composition (11, 16, 17). In coronary imaging, commonly used basis material pairs include water-iodine, water-calcium, and lipid-fibrous tissue, which provide the theoretical foundation for plaque composition analysis (12, 16).

### 2.2 Major technical implementations of spectral CT

In this review, spectral CT is used as an umbrella term for CT techniques that acquire or reconstruct energy-dependent attenuation information for material-specific or energy-resolved analysis. Conventional dual-energy CT represents the most widely implemented clinical form of spectral CT, whereas photon-counting CT represents a detector-level innovation that can provide both improved spatial resolution and intrinsic energy discrimination. Thus, PCCT should not be viewed as separate from spectral CT, but rather as an advanced implementation that extends spectral imaging from dual-energy material decomposition toward multi-threshold energy-resolved imaging with improved geometric fidelity.

In current clinical practice, spectral CT is represented primarily by dual-energy CT. Its principal technical implementations include dual-source CT, rapid kilovoltage switching, and dual-layer detector systems. Dual-source and rapid kilovoltage-switching approaches achieve spectral separation at the source or acquisition level, whereas dual-layer detector systems separate high- and low-energy photons at the detector level. Among these implementations, dual-layer detector CT has been widely used for coronary applications because it provides retrospectively available spectral data and relatively stable signal-to-noise performance (11, 18, 19).

PCCT differs conceptually from conventional dual-energy systems because it uses photon-counting detectors rather than energy-integrating detectors. By registering individual photons and sorting them into energy bins, PCCT can provide intrinsic spectral information while also reducing electronic noise and enabling smaller detector elements. These features are particularly relevant to coronary plaque imaging because they may improve lumen-plaque boundary delineation, reduce calcium-related blooming, stabilize quantitative plaque measurements, and support emerging K-edge molecular imaging (13, 14, 17). Nevertheless, PCCT remains an early clinical translation technology, with limited availability, platform-specific implementation, and incomplete outcome-based validation.

### 2.3 Core spectral imaging modes for coronary imaging

In clinical practice, spectral CT is used mainly through three imaging modes: virtual monoenergetic imaging, material decomposition imaging, and effective atomic number/electron density imaging (11, 17, 20). Low-keV virtual monoenergetic imaging enhances iodine contrast and improves delineation of the vessel-plaque interface. Material decomposition imaging is used to separate components such as iodine and calcium, thereby reducing artifacts. Effective atomic number (Zeff) and electron density imaging provide additional quantitative dimensions for tissue characterization (11, 20–22).

**Figure 1:**
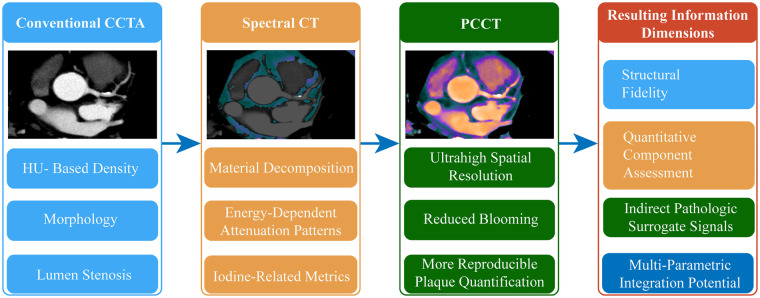
Evolution of coronary CT from density-based imaging to multidimensional plaque characterization. Conventional CCTA mainly provides density-based morphologic assessment and stenosis evaluation. Spectral CT introduces energy-resolved information for composition-sensitive plaque characterization, whereas photon-counting CT (PCCT) further improves spatial resolution, reduces blooming, and enhances quantitative reproducibility. These advances collectively expand coronary plaque assessment from conventional structural imaging toward multidimensional characterization.

The evolution from density-based coronary CT imaging toward multidimensional plaque characterization is schematically illustrated in Figure 1.

## 3 New findings of spectral CT in coronary plaque identification and their histopathologic correlates

### 3.1 From HU-based stratification to spectral information: expansion of the dimensionality of plaque composition characterization

Conventional CCTA has traditionally relied on CT attenuation stratification under a single spectral condition, together with visually defined high-risk features, to classify plaque type (8, 23). However, this approach remains fundamentally grounded in an empirical grayscale-threshold framework and is sensitive to scan parameters, tube voltage, reconstruction kernel, and calcification-related interference (12, 23). Because attenuation values overlap substantially among lipid-rich tissue, fibrous tissue, and mild calcification, a single HU value cannot reliably reflect the true composition of plaque (8, 23).

With the introduction of a multi-energy dimension, spectral CT has changed the basis of plaque identification. By analyzing attenuation patterns across different kiloelectron volt (keV) levels, or by extracting spectral histogram features from dual-layer detector systems, spectral CT enables characterization of energy-response patterns rather than reliance on a single density value within the same voxel (12, 24). In an intravascular ultrasound (IVUS)-referenced study, Mochizuki et al. showed that a feature model incorporating spectral information achieved discrimination between vulnerable and stable plaque, with an area under the curve of 0.81–0.85. This performance arose from the integrated expression of energy-dependent attenuation patterns rather than absolute HU differences alone (24).

From a histopathologic perspective, differences in effective atomic number and density between lipid-rich necrotic core and fibrous tissue result in distinct attenuation curves across energy levels. Spectral CT captures this relationship between composition and energy dependence (12, 25). Although direct measurement of fibrous cap thickness is still not possible, more stable estimation of lipid proportion and tissue heterogeneity provides an imaging surrogate that more closely reflects the material basis of plaque pathology (24, 25).

Current evidence remains largely exploratory. These studies demonstrate additional discriminatory information from the spectral dimension, but do not yet establish a mature threshold-based plaque classification system (24, 25).

### 3.2 From calcium obscuration to boundary reconstruction: correction of structural fidelity by ultrahigh-resolution PCCT

In conventional CCTA, calcified plaque often causes marked blooming artifact, obscuring the luminal boundary and leading to overestimation of stenosis severity (13). For many years, this phenomenon was regarded as an inherent limitation of CT spatial resolution. The emergence of PCCT has enabled this issue to be re-evaluated at the clinical level (13, 26).

In an intraindividual comparative study, Vecsey-Nagy et al. found that, compared with energy-integrating detector CT, ultrahigh-resolution photon-counting detector CT (UHR PCD-CT) measured significantly lower degrees of stenosis in calcified and partially calcified lesions and yielded values closer to those obtained with quantitative coronary angiography. Approximately 49% of patients showed a change in Coronary Artery Disease-Reporting and Data System (CAD-RADS) category (26). These findings indicate that part of the measurement error of conventional CCTA in calcified lesions is a directional bias that can be systematically corrected through improvements in detector technology and spatial resolution.

A 2025 Radiology study further showed that UHR PCD-CT provided markedly better reproducibility for measuring low-attenuation plaque volume than energy-integrating detector CT, with reader-to-reader intraclass correlation coefficients of 0.92 vs. 0.47 (27). This suggests that spectral CT changes not only what can be seen, but also whether it can be quantified reproducibly.

From a pathologic standpoint, calcification manifests across different stages and spatial scales. Macrocalcification is generally associated with fibrosis and relative plaque stability, whereas microcalcification may contribute to local fibrous cap stress concentration (28). Although current CT spatial resolution remains insufficient to directly resolve microcalcification, the improvements provided by UHR PCCT in boundary sharpening and volumetric estimation create the technical basis for more refined investigation of calcification patterns in the future.

### 3.3 From structural features to surrogate signals of pathobiologic processes: imaging correlates of iodine enhancement behavior and inflammation/microvasculature

Conventional CCTA primarily provides structural information, whereas the key pathobiologic processes underlying plaque instability, including inflammatory cell infiltration and neovascularization, are difficult to capture directly by structural features alone (7). Through iodine-based material decomposition and delayed-phase analysis, spectral CT provides a new approach for evaluating contrast behavior within the plaque itself (29).

Using delayed-enhancement dual-layer spectral CT and optical coherence tomography (OCT) as the reference standard, Nadjiri et al. found that conventional HU values could not distinguish high-risk from low-risk noncalcified plaques, whereas the minimum delayed iodine enhancement was significantly lower in high-risk plaques, approximately 1.0 mg/mL vs. 2.2 mg/mL (29). This finding suggests that intraplaque iodine distribution may reflect tissue permeability, microvascular density, or differences in tissue composition rather than luminal enhancement alone.

At a more advanced level, Si-Mohamed et al. used PCCT K-edge imaging combined with gold nanoparticles to achieve *in vivo* quantification of plaque macrophage burden in an animal model (30). Because macrophage infiltration is a key pathologic basis of active plaque inflammation and rupture risk, this study provided molecular-level proof that spectral CT has the physical capability to extend toward quantitative assessment of inflammation.

Delayed iodine enhancement and molecular imaging remain in early development. Their findings currently represent mechanistic validation rather than routine clinical application (17, 29, 30). Nevertheless, these studies suggest that spectral CT is evolving from structural identification toward an integrated imaging model that links structure with pathobiologic processes.

**Figure 2:**
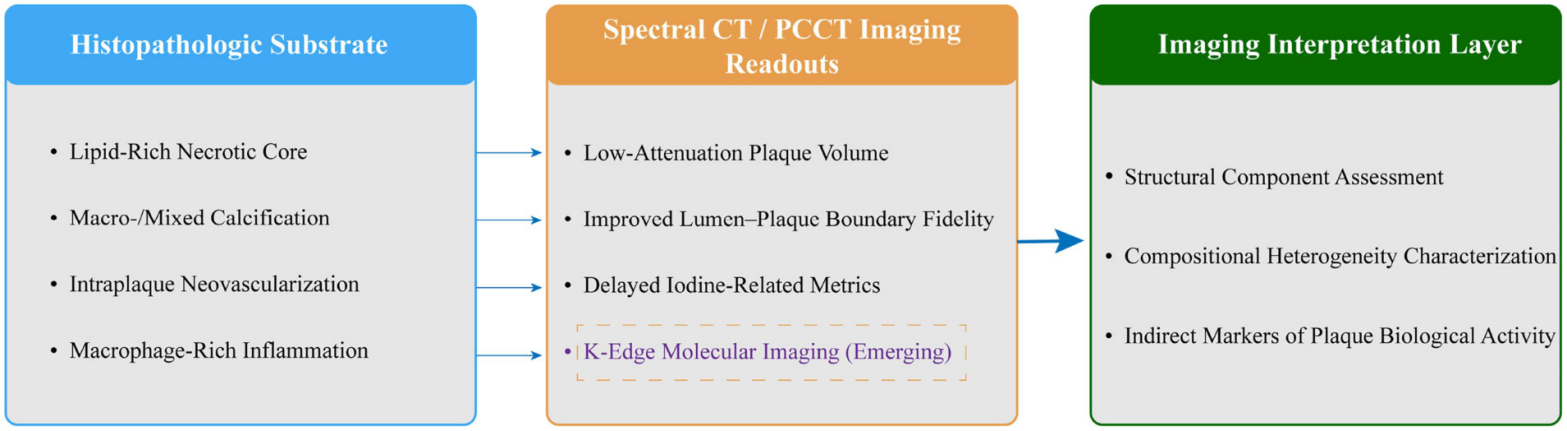
Imaging–pathology mapping in spectral and photon, counting CT plaque characterization. Spectral CT and photon-counting CT (PCCT) provide imaging readouts that can be linked, directly or indirectly, to major histopathologic features of coronary atherosclerotic plaque. Low-attenuation plaque volume serves as an imaging correlate of lipid-rich necrotic core burden, whereas improved lumen–plaque boundary fidelity is particularly relevant to calcified plaque assessment by reducing calcium-related boundary distortion. Delayed iodine-related metrics may reflect permeability-related or neovascularization-associated changes and thus provide indirect information on plaque pathobiologic activity. K-edge molecular imaging, although largely preclinical, illustrates the potential extension of PCCT toward inflammation-targeted molecular characterization.

In summary, recent advances in spectral CT for coronary plaque identification are characterized by expansion of the dimensions of plaque characterization and improvement in measurement stability. Multi-energy information and ultrahigh-resolution imaging bring plaque composition assessment, calcified boundary reconstruction, and extraction of selected surrogate signals of pathobiologic processes closer to their histologic basis, thereby extending the characterization capability of conventional CCTA (12, 26, 27). The major spectral CT- and PCCT-related imaging biomarkers discussed in this section are summarized in Table 1, and their broader imaging–pathology relationships are illustrated in Figure 2.

**Table 1:** Major spectral CT- and PCCT-related imaging biomarkers for coronary plaque characterization.

Biomarker	Technical basis	Pathological relevance and clinical value	Major limitations
Energy-dependent attenuation patterns	Attenuation variation across keV levels; spectral curves or histogram features	Different energy-response patterns may reflect lipid-rich, fibrous, and calcified components; enables plaque characterization beyond single-HU-based interpretation	Small studies; vendor/reconstruction dependence; no universal thresholds
Effective atomic number/electron density	Material-specific quantitative maps derived from spectral decomposition	Reflects differences in atomic composition and tissue density; may support tissue differentiation and supplementary plaque typing beyond HU-based classification	Limited coronary validation; sensitive to motion and partial-volume effects
Low-attenuation plaque volume	Material decomposition and HU threshold-based segmentation; reproducibility substantially improved with PCCT	Surrogate of lipid-rich necrotic core burden; may enable more reproducible quantification of high-risk plaque burden	Not equivalent to histology; threshold variability across protocols
Calcified plaque boundary fidelity/blooming reduction	Ultrahigh-resolution PCCT; smaller detector pixels; sharper delineation of the lumen-calcium interface	Macrocalcification is associated with fibrosis and relative plaque stability, whereas microcalcification may contribute to fibrous cap stress; improved boundary fidelity reduces stenosis overestimation and may improve downstream referral efficiency	Limited PCCT availability; evidence mainly from single-center or early clinical studies
Delayed iodine-related metrics	Iodine material decomposition and delayed-phase acquisition	May reflect permeability, neovascularization, or plaque microenvironment; provides a surrogate marker of high-risk plaque biology beyond morphology	Timing-sensitive; modest specificity; limited validation
K-edge molecular imaging	PCCT energy thresholding with high-Z contrast agents	Enables preclinical macrophage- or inflammation-targeted plaque imaging; illustrates the molecular imaging potential of PCCT	Currently limited to animal models; no clinically approved targeted contrast agents; translational pathway unresolved

CT, computed tomography; HU, Hounsfield unit; PCCT, photon-counting computed tomography; Z, atomic number.

## 4 Clinical significance of spectral CT in coronary plaque risk stratification

### 4.1 Risk reclassification in calcification-dominant lesions: From blooming-driven overestimation to anatomy-based correction

The technical limitations described in Section 3.2 have direct implications for clinical risk stratification. In patients with a high calcific burden, conventional CCTA may systematically overestimate stenosis severity, thereby affecting CAD-RADS classification and downstream referral decisions.

A 2024 Radiology study showed that, as spatial resolution improved, diameter stenosis in calcified lesions decreased progressively, falling from 41.5% to 26.7% in the *in vivo* analysis and resulting in downward reclassification of CAD-RADS category in more than half of patients (54.4%) (13). An intraindividual comparative study published in Circulation: Cardiovascular Imaging in 2024 further confirmed that PCD-CT measured significantly lower stenosis in calcified and partially calcified plaques than energy-integrating detector CT, leading to category changes in 49% of patients (26). Importantly, this difference was concentrated mainly in calcification-related lesions and was not significant in noncalcified plaques (26).

From the perspective of risk stratification, these findings imply that, in calcification-dominant populations, risk classification by conventional CCTA may be driven in part by imaging artifact, whereas PCCT, by improving spatial resolution and boundary reconstruction, reduces this systematic bias. Its value lies not in simply lowering the category, but in making classification more closely reflect the underlying anatomy (13, 26, 27).

Although these studies used quantitative coronary angiography or invasive coronary angiography as reference standards, sample sizes remained relatively small and most data were derived from single-center platforms (13, 21, 26, 27, 31). A rigorous conclusion is that PCCT shows a directionally consistent corrective effect in calcification-related stenosis measurement, but its independent impact on long-term event-based risk stratification still requires validation in higher-level studies (14).

### 4.2 Reproducible quantification of high-risk burden: stratification potential of low-attenuation plaque and iodine-related parameters

Unlike stenosis severity, plaque composition and tissue heterogeneity are more directly linked to the underlying pathology. In risk stratification, the potential incremental value of spectral CT is reflected mainly in two aspects: more stable and reproducible quantification of high-risk compositional burden, and supplementary surrogate signals, such as iodine-related parameters, that may reflect underlying pathobiologic processes (12, 14).

At the level of structural burden, conventional CCTA shows limited reproducibility in the measurement of low-attenuation plaque volume, restricting its stable incorporation into risk stratification models (10, 23). An intraindividual comparative study published in Radiology in 2025 showed that UHR PCD-CT was markedly superior to EID-CT in quantifying low-attenuation plaque volume, with substantially higher interreader agreement (ICC, 0.92 vs 0.47) (27). This finding suggests that low-attenuation burden may shift from a high-noise variable to a more reproducible quantitative metric under PCCT conditions.

At the level of surrogate signals of pathobiologic processes, delayed iodine-enhancement parameters provide information distinct from that of structural burden alone. Nadjiri et al. showed that delayed-phase iodine uptake was significantly lower in high-risk plaques, whereas conventional HU values had no discriminatory value (29). This suggests that, in some settings, spectral parameters may capture differences in the tissue microenvironment that are not reflected by conventional structural markers.

An increase in low-attenuation volume does not automatically mean that the measurement is closer to pathologic truth; its correspondence with histology requires further validation through invasive imaging or pathologic correlation studies. Likewise, delayed iodine enhancement is limited by small samples, modest specificity, scan timing, and contrast kinetics. At present, spectral CT is best interpreted as providing two levels of information for risk stratification: more stable quantification of high-risk compositional burden and supplementary surrogate signals that may reflect selected pathobiologic processes (12, 14, 23).

### 4.3 Real-World pathway optimization: improving positive referral yield rather than simply increasing detection

The ultimate value of risk stratification lies in its ability to alter clinical pathways. Studies published over the past 5 years have shown a consistent signal that PCCT improves diagnostic specificity and positive predictive value (PPV) (14, 32).

A 2024 study published in the Journal of Cardiovascular Computed Tomography showed that, compared with conventional wide-coverage EID-CT, PCD-CT significantly reduced referral rates for subsequent invasive coronary angiography, with the difference being more pronounced in patients with a high calcific burden (33). In a larger propensity score-matched study, the specificity for detecting significant stenosis increased from 0.74 to 0.81 in the PCD-CT group, and the proportion of invasive angiograms not followed by revascularization was reduced (3.7% vs 8.0%) (34). A 2025 Journal of the American College of Cardiology study of consecutive patients further showed that, among 7,833 patients, the PCD-CT group had a lower referral rate for invasive angiography, while the proportion undergoing revascularization among those referred was higher; at the vessel level, specificity and PPV were significantly higher with PCD-CT than with EID-CT, whereas sensitivity and negative predictive value remained comparable (35).

Taken together, this body of evidence suggests that the most consistent near-term value of spectral CT and PCCT lies in improved calcified stenosis assessment, more reproducible plaque quantification, and better downstream referral efficiency (13, 26, 27, 33–35). For PCCT in particular, the clearest incremental value appears to be reducing unnecessary invasive testing and optimizing positive downstream referrals rather than simply increasing detection rates (33–35). However, these findings should still be interpreted as a consistent signal from current studies rather than definitive prognostic evidence validated at the level of clinical endpoints. The key clinical and translational studies supporting these conclusions are summarized in Table 2.

**Table 2:** Key clinical and translational studies of spectral CT and PCCT in coronary plaque assessment.

Study	Year	Modality/platform	Design/reference standard	Main target	Key quantitative findings	Main implication	Limitation
Mochizuki et al.	2024	Spectral CT + machine learning	IVUS-referenced study	Vulnerable plaque discrimination	AUC 0.81 to 0.85	Spectral information improves discrimination beyond HU	Small-scale; limited model validation
Nadjiri et al.	2022	Delayed-enhancement dual-layer spectral CT	OCT reference	High-risk noncalcified plaque	Minimum delayed iodine: 1.0 mg/mL (high-risk) vs 2.2 mg/mL (low-risk)	Iodine metrics may reflect plaque microenvironment	Small sample; timing-sensitive
Halfmann et al.	2024	UHR PCCT	Clinical stenosis assessment	Calcified stenosis	Diameter stenosis decreased from 41.5% to 26.7%; CAD-RADS down-classification in 54.4% of patients	Blooming correction and stenosis reclassification	Single-platform validation
Vecsey-Nagy et al.	2024	UHR PCD-CT vs EID-CT	Intraindividual comparison; QCA/ICA reference	Calcified stenosis	49% CAD-RADS category change	More accurate calcified lesion assessment	Sample size and center limitations
Vecsey-Nagy et al.	2025	UHR PCD-CT vs EID-CT	Intraindividual comparison	Plaque quantification	ICC 0.92 vs 0.47 for LAP volume	Improved reproducibility of plaque burden	Histologic validation lacking
Simon et al.	2024	PCD-CT vs EID-CT	Downstream ICA referral analysis	Clinical pathway	Significantly reduced ICA referral rate, especially in patients with high calcific burden	Better positive referral yield	Retrospective design
Nakashima et al.	2025	PCD-CT vs EID-CT	Retrospective propensity-matched study	Stable coronary artery disease	Specificity increased from 0.74 to 0.81; non-revascularized ICA decreased from 8.0% to 3.7%	Improved diagnostic specificity and reduced unnecessary invasive testing	Retrospective design; single-center; generalizability to unselected populations uncertain
Sakai et al.	2025	PCD-CT vs EID-CT	Consecutive patient cohort	Diagnostic performance and clinical impact	Higher specificity and PPV; comparable sensitivity and NPV	Reduced false positives and improved referral efficiency	Outcome validation still needed

CAD-RADS, Coronary Artery Disease-Reporting and Data System; EID-CT, energy-integrating detector CT; HU, Hounsfield unit; ICA, invasive coronary angiography; ICC, intraclass correlation coefficient; IVUS, intravascular ultrasound; LAP, low-attenuation plaque; NPV, negative predictive value; OCT, optical coherence tomography; PCD-CT, photon-counting detector CT; PCCT, photon-counting computed tomography; PPV, positive predictive value; QCA, quantitative coronary angiography; UHR, ultrahigh-resolution.

## 5 Comparison of spectral CT with other cardiovascular imaging modalities

### 5.1 Complementary roles relative to IVUS and OCT: differences in imaging principles and clinical positioning

The distinction between spectral CT and IVUS/OCT does not primarily lie in which modality is more advanced, but rather in their fundamentally different principles of information acquisition and imaging scale. IVUS and OCT are intravascular imaging techniques in which the probe is placed within the coronary lumen, allowing near-field acquisition of vessel wall and plaque information (36, 37). IVUS is based on ultrasound backscatter signals and is well suited for assessing overall plaque burden, vascular remodeling, and the extent of the external elastic membrane. OCT is based on near-infrared optical coherence principles and offers higher axial resolution, enabling direct visualization of fibrous cap thickness, superficial microcalcification, and selected inflammation-related signals (36–38).

By contrast, spectral CT is an extravascular tomographic technique. Its principal advantage does not come from proximity to the lesion, but from the ability to use the energy dependence of x-ray attenuation to achieve noninvasive assessment of structure and composition across the entire coronary tree. Even under UHR PCCT, its spatial resolution remains lower than that of OCT; therefore, it cannot directly visualize the true morphology of the fibrous cap or microcalcification. However, it can provide relatively stable information on plaque burden, low-attenuation components, and calcified plaque boundaries across the entire coronary tree (13, 26, 27).

These differences define distinct clinical roles. IVUS and OCT are better suited for focal, high-detail evaluation in interventional settings, such as clarifying lesion morphology, guiding stent strategy, and confirming fibrous cap rupture or local microstructural abnormalities (36, 37). Spectral CT is noninvasive, covers the entire coronary tree, and is better suited for assessment of coronary plaque burden, correction of stenosis overestimation in calcification-dominant lesions, and longitudinal follow-up. Thus, the appropriate clinical interpretation is complementarity rather than replacement.

### 5.2 Multimodality integration with PET/CT and CMR: differences in information dimensions and complementary clinical positioning

Compared with spectral CT, positron emission tomography/computed tomography (PET/CT) and cardiac magnetic resonance (CMR) provide pathologic information at different levels. PET/CT relies on specific tracers to reflect molecular or metabolic processes such as inflammation and active calcification. Its major strength lies in revealing biologic activity, but precise localization and quantification of coronary plaque remain challenging because of limited spatial resolution, cardiac motion, and background tracer uptake (39–41). CMR primarily assesses vessel wall inflammation, fibrosis, and regional functional changes on the basis of tissue signal characteristics. It avoids ionizing radiation and offers excellent soft-tissue contrast, but its spatial resolution and technical maturity remain limited for imaging small, rapidly moving coronary arteries (42, 43).

Spectral CT occupies a different position from both modalities. It remains a high-resolution tomographic technique based on anatomic imaging, while using multi-energy information to provide composition-related parameters and selected surrogate signals of pathobiologic processes (42, 43). PET/CT emphasizes molecular activity, CMR emphasizes tissue characteristics and functional context, whereas spectral CT is better suited to establishing a quantitative structural-compositional framework across the entire coronary tree (42, 43).

From a clinical perspective, spectral CT is better positioned as the structural-compositional platform within a multimodality assessment strategy, with PET/CT providing complementary molecular activity information and CMR contributing tissue and functional information (39, 42, 43). The principal barrier to multimodality integration lies in the lack of uniformity in parameter definitions, spatial coregistration, and quantitative standards across modalities. To clarify the distinct yet complementary roles of spectral CT, PCCT, IVUS, OCT, PET/CT, and CMR, their principal information domains, strengths, limitations, and current clinical positioning are summarized in Table 3.

**Table 3:** Comparative roles of coronary imaging modalities in plaque assessment.

Modality	Primary information domain	Main strength	Main limitation	Best-suited clinical role	Current role in plaque assessment
Spectral CT	Whole-vessel structural and compositional assessment	Material decomposition; energy-resolved characterization	Limited standardization; cross-platform variability; indirect tissue interpretation	Noninvasive plaque characterization	Adjunctive compositional imaging modality
Photon-counting CT (PCCT)	Whole-vessel structural imaging with improved quantitative fidelity	Higher spatial resolution; reduced blooming; reproducible plaque quantification	Limited availability; platform dependence; post-processing burden; incomplete outcome validation	Assessment of calcified lesions and plaque burden	Noninvasive plaque imaging tool in early clinical translation
IVUS	Local plaque burden and vessel wall structure	Deep penetration; remodeling assessment	Invasive; limited superficial resolution	PCI planning and intraprocedural assessment	Reference tool for plaque burden and remodeling
OCT	Local plaque microstructure	High-resolution fibrous cap and microstructure assessment	Invasive; limited penetration	Plaque rupture detection and PCI guidance	Reference tool for plaque microstructure
PET/CT	Molecular and metabolic activity	Assessment of inflammatory activity and active microcalcification	Limited spatial resolution; difficult coronary localization	Research assessment of plaque activity	Complementary molecular imaging modality
CMR	Tissue characterization and functional context	Excellent soft-tissue contrast; no ionizing radiation	Limited spatial resolution for coronary plaque imaging	Vessel wall and functional assessment	Complementary tissue and functional imaging modality

CMR, cardiac magnetic resonance; CT, computed tomography; IVUS, intravascular ultrasound; OCT, optical coherence tomography; PCI, percutaneous coronary intervention; PCCT, photon-counting computed tomography; PET/CT, positron emission tomography/computed tomography.

.

## 6 Challenges, limitations, and future directions

### 6.1 Technical and methodologic challenges: physical boundaries of spatial scale, motion control, and spectral stability

Despite important progress in coronary plaque characterization, spectral CT remains constrained by the fundamental physical conditions of cardiovascular imaging (11, 44). First, the rapid cyclic motion of the coronary arteries continues to challenge temporal resolution and motion-correction algorithms. Extraction of multi-energy information depends on precise voxel-to-voxel correspondence, and any motion artifact or reconstruction error may be amplified at the level of material decomposition, thereby affecting the stability of spectral parameters. This issue is particularly pronounced in patients with irregular heart rates or rhythm variability (44–46).

Second, spatial resolution remains a central limitation for detailed characterization of coronary plaque. Even under UHR PCCT, the imaging scale remains submillimeter and therefore differs by orders of magnitude from micrometer-scale techniques such as OCT. As a result, the compositional information currently provided by spectral CT essentially represents a voxel-averaged expression, making it difficult to directly depict microstructural features such as fibrous cap thickness or clusters of microcalcification. Spectral CT is therefore better suited for assessment of macroscopic compositional burden than for microstructural tomographic analysis (11, 37).

Third, there is an inherent trade-off between spectral stability and dose control. Although low-keV virtual monoenergetic imaging can enhance contrast, it is often accompanied by increased noise. Material decomposition algorithms are highly sensitive to signal-to-noise ratio and are therefore more vulnerable in low-dose settings or in patients with larger body habitus. Future progress will depend not only on hardware optimization, but also on iterative reconstruction, noise modeling, and multi-energy data fusion algorithms (11, 47).

### 6.2 Quantitative standardization and clinical translation: the Gap between parameter generation and generalizable threshold systems

Compared with physical limitations, an even greater challenge lies in quantitative standardization and cross-platform consistency (14, 23). The spectral parameters generated by current spectral CT systems, including low-attenuation volume, iodine concentration, effective atomic number, and spectral attenuation curve slope, vary across vendor platforms, detector designs, reconstruction kernels, and material decomposition algorithms (10, 14, 23). Even within the same platform, different keV selections and decomposition strategies may alter quantitative results (10, 14, 20). This methodologic heterogeneity makes it difficult to compare findings across centers and hampers the establishment of unified thresholds (23, 48).

Among spectral parameters, iodine concentration, low-keV VMI attenuation, spectral curve slope, and delayed iodine-related metrics are particularly susceptible to inter-platform variability because they depend strongly on energy separation, contrast timing, reconstruction kernel, noise suppression, and material decomposition algorithms. Low-attenuation plaque volume is additionally affected by spatial resolution, partial-volume effects, lumen segmentation, and HU threshold definitions. Effective atomic number and electron density maps may offer more quantitative information, but their coronary application remains sensitive to motion, calibration, and vendor-specific implementation. Future standardization should therefore prioritize harmonized acquisition protocols, keV level selection, reconstruction kernels, plaque segmentation rules, material decomposition algorithms, phantom calibration, and multicenter reproducibility testing.

Current evidence regarding high-risk parameters is derived mainly from single-center studies or specific device platforms, with a lack of large-scale, prospective, multi-platform validation (14, 20). Although metrics such as low-attenuation plaque volume and delayed iodine enhancement have clear mechanistic plausibility, there is still no consensus regarding optimal thresholds, reproducibility limits, or minimum clinically important differences (10, 11, 20). Without explicit quantitative standards, these parameters will remain difficult to incorporate into clinical guidelines or routine reporting systems (23, 48).

### 6.3 Real-World clinical implementation: dose, access, workflow, and reporting

From a real-world perspective, the most defensible near-term role of spectral CT and PCCT is not routine replacement of standard CCTA in all patients, but selective use in scenarios where conventional CCTA is known to be vulnerable, such as heavily calcified coronary arteries, uncertain stenosis severity, or discordance between anatomic findings and clinical probability. Current evidence supports improved structural fidelity, reproducibility, and positive referral yield, but remains insufficient to justify broad implementation for endpoint-based risk prediction. Future studies should evaluate whether spectral CT-derived parameters change reporting confidence, downstream testing, treatment selection, and patient outcomes in routine clinical pathways.

Radiation dose remains an important implementation issue. Although some spectral and PCCT protocols may maintain dose efficiency through improved detector performance and reconstruction algorithms, low-keV VMI, multiphase delayed iodine imaging, or repeated reconstructions may increase noise-management demands or acquisition complexity. Future clinical studies should report effective dose, contrast volume, heart-rate control, reconstruction settings, and post-processing time alongside diagnostic performance.

Scanner availability and cost are also major barriers. PCCT systems remain concentrated in specialized centers and are not yet widely available in routine coronary CT practice. Even when spectral datasets are acquired, their clinical value depends on access to validated post-processing software, trained readers, and standardized reporting templates.

Workflow integration should be considered a key translational endpoint. Spectral analysis may require additional segmentation, material-map reconstruction, keV optimization, plaque component quantification, and quality control. These steps may increase interpretation time and require specific expertise. For routine adoption, spectral CT biomarkers must be simplified into robust, reproducible, and clinically actionable outputs rather than a large set of exploratory parameters.

### 6.4 Future directions: synergistic advances in hardware, algorithmic integration, and multimodality imaging

Despite the challenges outlined above, the developmental trajectory of spectral CT has become increasingly clear (14, 32). First, PCCT represents a major advance in detector technology. Its energy-discrimination capability and smaller pixel size provide the technical basis for reducing calcium-related blooming, improving the stability of low-attenuation component quantification, and exploring K-edge molecular imaging (30, 32). As system maturity and dose optimization continue to improve, its application in coronary imaging is likely to expand further (14, 32).

Second, artificial intelligence and radiomics provide a methodological framework for integrating multidimensional spectral data. Spectral CT inherently generates multi-energy, multiparametric datasets that extend beyond conventional single-energy CCTA. By integrating structural, compositional, and spectral-response features through machine learning models, it may become possible to build more expressive frameworks for risk characterization (49, 50). However, model generalizability depends on multicenter data and standardized annotation, and algorithms lacking external validation are unlikely to achieve meaningful clinical implementation (49–51).

Third, multimodality integration will likely become an important future direction. The strengths of spectral CT in structural fidelity and quantitative compositional assessment are naturally complementary to the molecular activity information provided by PET and the tissue and functional information provided by CMR. A precise assessment framework may depend less on a breakthrough in any single technology than on the integration of multimodal data within a unified risk-representation model (39, 50, 51).

In the long term, the core value of spectral CT may lie not in optimization of any single parameter, but in the development of integrated models linking structure, composition, and surrogate functional signals, followed by incorporation into clinical decision-making pathways after standardization and validation have been achieved (14, 39, 49).

## 7 Conclusion

Spectral CT is reshaping the imaging characterization of coronary plaque. Compared with conventional CCTA, its principal advance lies not simply in generating more parameters, but in improving structural fidelity and the stability of compositional quantification through multi-energy information and higher spatial resolution, while also expanding, to a certain extent, the ability to capture surrogate signals of underlying pathobiologic processes (14, 32).

However, current evidence remains insufficient to conclude that these technical advantages have translated into robust benefits at the level of clinical endpoints. Its independent incremental value for long-term event prediction, the consistency of parameters across platforms, the establishment of generalizable threshold systems, and feasibility in routine clinical workflows all remain inadequately validated (14, 23). Compared with IVUS/OCT, PET/CT, and CMR, spectral CT is better understood as a noninvasive platform for integrated structural-compositional assessment rather than as a direct substitute for microstructural or molecular imaging.

Looking forward, the clinical translation of spectral CT will depend mainly on progress in four areas: further optimization of hardware and reconstruction techniques, establishment of cross-platform quantitative standardization, practical workflow integration, and validation of its value within risk models through multicenter prospective studies (14, 23, 39). On this basis, spectral CT may evolve from a coronary imaging technique focused primarily on anatomic depiction into an important component of precise coronary plaque assessment.
